# Sam68 is required for the growth and survival of nonmelanoma skin cancer

**DOI:** 10.1002/cam4.2513

**Published:** 2019-08-22

**Authors:** Kai Fu, Xin Sun, Xue Xia, Ryan P. Hobbs, Yajuan Guo, Pierre A. Coulombe, Fengyi Wan

**Affiliations:** ^1^ Institute of Molecular Precision Medicine and Hunan Key Laboratory of Molecular Precision Medicine Xiangya Hospital Central South University Changsha Hunan China; ^2^ Department of Oncology Xiangya Hospital Central South University Changsha Hunan China; ^3^ Department of Biochemistry and Molecular Biology Bloomberg School of Public Health Johns Hopkins University Baltimore MD USA; ^4^ The Rockefeller University New York NY USA; ^5^ Department of Dermatology College of Medicine Pennsylvania State University Hershey PA USA; ^6^ Department of Cell and Developmental Biology University of Michigan Medical School Ann Arbor MI USA; ^7^ Department of Dermatology University of Michigan Medical School Ann Arbor MI USA; ^8^ Department of Oncology Johns Hopkins University School of Medicine Johns Hopkins Medical Institutions Baltimore MD USA; ^9^ Sidney Kimmel Comprehensive Cancer Center Johns Hopkins Medical Institutions Baltimore MD USA; ^10^ W. Harry Feinstone Department of Molecular Microbiology and Immunology Bloomberg School of Public Health Johns Hopkins University Baltimore MD USA

**Keywords:** DNA damage responses, NF‐κB, Sam68, skin cancer

## Abstract

Although targeting DNA repair signaling pathways has emerged as a promising therapeutic for skin cancer, the relevance of DNA damage responses (DDR) in the development and survival of nonmelanoma skin cancer (NMSC), the most common type of skin cancer, remains obscure. Here, we report that Src‐associated substrate during mitosis of 68 kDa (Sam68), an early signaling molecule in DDR, is elevated in skin tumor tissues derived from NMSC patients and skin lesions from *Gli2*‐transgenic mice. Downregulation of Sam68 impacts the growth and survival of human tumor keratinocytes and genetic ablation of Sam68 delays the onset of basal cell carcinomas (BCC) in *Gli2*‐transgenic mice. Moreover, Sam68 plays a critical role in DNA damage‐induced DNA repair and nuclear factor kappa B (NF‐κB) signaling pathways in keratinocytes, hence conferring keratinocyte sensitivity to DNA damaging agents. Together, our data reveal a novel function of Sam68 in regulating DDR in keratinocytes that is crucial for the growth and survival of NMSC.

## Graphical abstract

Here, we report that Sam68, an early signaling molecule in DDR, is elevated in skin tumor tissues derived from NMSC patients and skin lesions from Gli2‐transgenic mice. Downregulation of Sam68 impacts the growth and survival of human tumor keratinocytes and genetic ablation of Sam68 delays the onset of basal cell carcinomas (BCC) in Gli2‐transgenic mice. Moreover, Sam68 plays a critical role in DNA damage‐induced DNA repair and nuclear factor kappa B (NF‐κB) signaling pathways in keratinocytes, hence conferring keratinocyte sensitivity to DNA damaging agents.

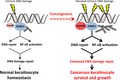

## 1 INTRODUCTION

Skin cancer is the most commonly diagnosed malignancy in the United States, and the incidence of skin cancer has increased dramatically over the last few decades.^1^ Nonmelanoma skin cancer (NMSC), which includes squamous cell carcinomas (SCCs) and basal cell carcinomas (BCCs), is the most common type of skin cancer and is associated with significant morbidity and mortality.^2^, ^3^ It has been well documented that DNA damage frequently caused by irradiation in keratinocytes and melanocytes plays a crucial role in the initiation and progression of skin cancer in high‐risk individuals 4, 5; however, the relevance of DNA damage responses (DDR) in tumor skin keratinocytes has not been extensively investigated yet.

Skin cancer cells, similar to other types of cancer cells, are continuously challenged by intrinsic stresses including the accumulation of replication errors in rapidly proliferating cells that could ultimately add to the DNA damage burden.^6^ In order to conquer this intrinsic DNA damage and avoid the programmed cell death associated with it, cancer cells generally acquire an elevated DNA repair capability through a hyperactivated DDR machinery.^7^ Targeting DDR processes can induce antitumor effects when used as a monotherapy or in combination with other therapies in various tumor settings.^8^, ^9^ In particular, recent studies have shown signs of promise when inhibiting poly(ADP‐ribose) polymerase 1 (PARP1) conferred DDR in melanoma.^10^, ^11^ Whether DDR plays an important role in the development and survival of NMSC and whether it could be therapeutically targeted in NMSC remain largely unknown. Our recent studies reveal that Src‐associated substrate during mitosis of 68 kDa (Sam68) is an early‐onset and key effector of the response to DNA damage.^12^, ^13^, ^14^ Elevated expression of Sam68 occurs in multiple types of cancers and also correlates with tumor progression.^14^, ^15^, ^16^, ^17^, ^18^ Sam68 is ubiquitously expressed in many tissues including the skin 19; however, the significance of Sam68 in skin cancer development and survival has not yet been examined.

In this paper, we show that the steady‐state levels of Sam68 are elevated in the skin tumors derived from human BCC and SCC patients and in skin tumor lesions from genetically manipulated *Gli2* ^tg/+^ mice. Ablation of Sam68 dramatically delays the spontaneous onset of tumor lesions in *Gli2* ^tg/+^ mouse ears. Moreover, Sam68 knockdown attenuates DNA damage‐induced DNA repair and NF‐κB signaling pathways, suggesting a key role of Sam68 in DDR in keratinocytes. Collectively, our findings reveal a crucial role of Sam68 in the development and survival of NMSC through regulating multiple signaling pathways in DDR.

## 2 MATERIALS AND METHODS

### 2.1 Ethics statement

All animal experiments were performed according to protocol number MO16H285, approved by the Johns Hopkins University's Animal Care and Use Committee and in direct accordance with the NIH guidelines for housing and care of laboratory animals. Human skin cancer tissue arrays were purchased from US Biomax Inc, where all human tissues have been collected under HIPAA‐approved protocols with donors being informed in full and with their consent. All samples tested negative for HIV and Hepatitis B or their counterparts in animals, and approved for commercial product development (see Table S1 for additional details).

### 2.2 Mice

*Sam68* ^−/−^ 14 and *Gli2* ^tg/+^ 20 mice in C57Bl/6 background were described previously. *Gli2* ^tg/+^; *Sam68* ^±^ mice were bred with *Sam68* ^±^ mice to generate the progeny of interest. All mice were maintained in a specific pathogen‐free facility and fed autoclaved food and water ad libitum.

### 2.3 Cell culture, antibodies, and reagents

Immortalized mouse epidermal keratinocytes cells and A431 human malignant keratinocytes, regularly tested for mycoplasma contamination, were cultured as described previously.^20^, ^21^ Antibodies used were: IκBα, Sam68, p65 from Santa Cruz Biotechnology; β‐actin from Sigma‐Aldrich; PECAM‐1 from Chemicon; PAR from Trevigen; phospho‐p65, Chk1, phospho‐Chk1, Chk2, Histone H3, phospho‐H3 from Cell Signaling Technology; phospho‐Chk2 from Novus Biologicals; 4′,6‐diamidino‐2‐phenylindole (DAPI) was obtained from Sigma‐Aldrich. Human tissue arrays containing skin tissue samples derived from healthy controls and squamous and basal cell carcinoma patients were purchased from US Biomax Inc.

### 2.4 Flow cytometry

For flow cytometry, cells were washed twice with PBS, resuspended in staining buffer (1% fetal bovine serum in PBS), and stained with annexin V/propidium iodide. Following staining and extensive washes with staining buffer, cells were analyzed on a FACSCalibur (BD Biosciences). Events were collected and analyzed with the FlowJo software (Tree Star).

### 2.5 γ‐Irradiation

γ‐Irradiation of A431 cells and immortalized keratinocytes was performed using a ^137^Cesium source (dose rate 8 Gy/min) as previously described.^13^

### 2.6 RNA interference and transfection

Mouse Sam68 siGENOME SMARTpool siRNA was purchased from Thermo Scientific. Human Sam68 siRNAs were described previously.^22^ Transient transfection of siRNAs into mouse/human keratinocytes was performed with Lipofectamine RNAiMAX (Life Technologies) according to the manufacturer's instructions.

### 2.7 Cell survival assays

After knockdown of Sam68 for 72 hours, 1 × 10^3^ of cells were γ‐irradiated at the indicated dose and immediately seeded in six‐well plate. After incubation for an additional 96 hours, surviving cells were counted using a Z1 Coulter Particle Counter (Beckman Coulter), and the survival fraction was calculated by normalizing the live cell numbers from indicated samples to that in nonradiated si‐NC cells.

### 2.8 Anchorage‐independent growth in soft agar

5 × 10^4^ cells suspended in 750 μL of DMEM containing 20% FBS were combined with 750 μL of 0.6% (w/v) agarose. This 0.3% agar/cell solution was plated on top of a 2‐ml layer of 0.5% agarose in one well on a six‐well plate. After 5 minutes, 500 μL of DMEM was added to cover the solidified agar/cells, and replaced with fresh medium every 3 days to prevent wells from drying out. Three weeks later, colonies were obtained using the FluorChem Q imaging system, and light intensity was adjusted to specifically show colonies in dark shades. Images were then processed using the ImageJ software (National Institutes of Health) to quantify colony number and diameter.

### 2.9 Immunoblot

Immunoblot assays were conducted as previously described.^23^ In brief, cells were harvested and lysed on ice by 0.4 mL of lysis buffer (50 mmol/L Tris‐HCl [pH 8.0], 150 mmol/L NaCl, 1% NP‐40, and 0.5% sodium deoxycholate, 1× complete protease inhibitor cocktail [Roche Applied Science]) for 30 minutes. The proteins were separated by sodium dodecyl sulfate‐polyacrylamide gel electrophoresis and transferred onto nitrocellulose membranes and probed by the Super Signaling system (Thermo Scientific) according to the manufacturer's instructions, and imaged using a FluorChem E System (Protein Simple).

### 2.10 Histology and immunohistology

After euthanizing mice, ear tissue was removed under aseptic conditions, fixed in 10% buffered formalin for 24 hours, embedded in paraffin and 5‐micron sections were then cut and processed for Hematoxylin and Eosin (H&E) staining. For immunohistology, after euthanizing mice, ear tissue was excised under aseptic conditions and frozen in optimal cutting temperature (OCT) media (Tissue‐Tek). Five micron frozen sections were cut using a Microm HM 550 Cryostat (Thermo Scientific), collected on coated slides, fixed in paraformaldehyde, washed with PBS, and blocked with appropriate sera in PBS. After incubating with appropriate antibodies, sections were washed and incubated with fluorescence dye‐conjugated second antibodies and 1 µg/mL of DAPI. Stained sections were washed and mounted under a coverslip using Fluoro‐gel with Tris Buffer (Electron Microscopy Sciences) and examined using a DMi8 fluorescent microscope (Leica). The measurement of ear thickness was carried out as described.^20^

### 2.11 Statistical analyses

All statistical analyses were performed using GraphPad Prism version 6.0 (GraphPad Software). Standard errors of the mean (SEM) were plotted in graphs. Differences are reported as follows: ns, nonsignificant difference; * different at *P* < .05; ** at *P* < .01; *** at *P* < .001; **** at *P* < .0001 by unpaired Student's *t* test.

## 3 RESULTS

### 3.1 Sam68 protein levels are elevated in human and mouse nonmelanoma skin cancer

**Figure 1:**
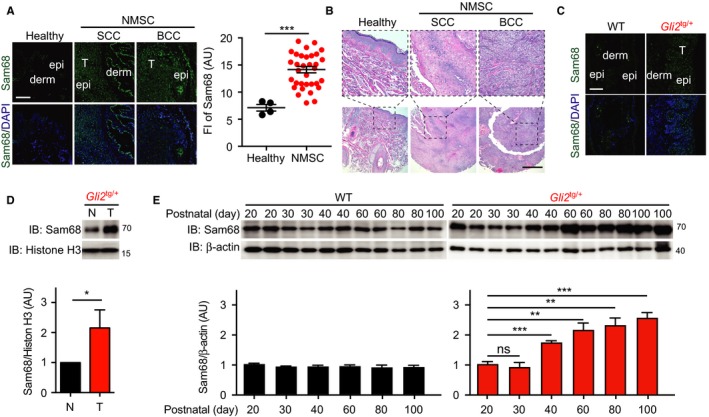
Sam68 protein level is elevated in human and mouse skin cancers. A, Immunofluorescence micrographs of Sam68, with nuclei counterstained by DAPI, on skin tissue sections from healthy controls or nonmelanoma skin cancer (NMSC) patients consisting of both squamous cell carcinoma (SCC) and basal cell carcinoma (BCC) cases. T, tumor; derm, dermis; epi, epidermis. Scale bars, 100 μm. *Right*, the fluorescence intensity (FI) of Sam68 on the indicated skin tissue sections was quantified. B, Hematoxylin and eosin staining of human healthy control skin, SCC, and BCC tissues. Scale bars, 500 μm. C, Immunofluorescence micrographs of Sam68, with nuclei counterstained by DAPI, on normal and tumor skin tissue sections from wild‐type (WT) and tumor‐laden *Gli2* ^tg/+^ mice, respectively. T, tumor; derm, dermis; epi, epidermis. Scale bars, 50 μm. D, Whole cell lysates derived from adjacent normal (N) and tumor (T) skin tissue from the same tumor‐laden *Gli2* ^tg/+^ mice at postnatal day 60 were immunoblotted (IB) for Sam68, with Histone H3 as a loading control. *Bottom*, normalized Sam68 level in the N and T skin tissues in *Gli2* ^tg/+^ mice (n = 3) was quantified. E, Skin tissue was collected from wild‐type mice or *Gli2* ^tg/+^ mice at the indicated postnatal days and whole cell lysates were derived (each sample indicates individual mouse) and IB for Sam68, with β‐actin as a loading control. The normalized Sam68 level in the skin of indicated mice was quantified on the bottom. Protein expression levels were measured from at least three mice per genotype/time point. AU, arbitrary unit. Data information: In (A, D‐E), data are presented as mean ± SEM. *P < .05; **P < .01; and ***P < .001 (Student's *t* test)

To assess the potential relevance of Sam68 in NMSC, we first examined Sam68 expression in human skin tissue samples derived from either healthy controls or NMSC patients. The protein levels of Sam68 were substantially elevated in the skin tissue from 16 BCC and 17 SCC patients, when compared to those in normal skin tissue from healthy controls (Figure 1A,B). We further measured Sam68 protein levels in skin tissue derived from *Gli2*‐transgenic (*Gli2* ^tg/+^) mice, which spontaneously develop basaloid follicular hamartoma lesions between postnatal 45 days (P45) and P120 that then become more like nodular BCC observed in human BCC patients by around P180.^20^, ^24^ Consistently, Sam68 was upregulated in lesions in the ear skin derived from *Gli2* ^tg/+^ mice, compared to the normal skin tissue derived from wild‐type (WT) mice (Figure 1C) or the adjacent normal tissue from the same tumor‐laden *Gli2* ^tg/+^ mice (Figure 1D). Moreover, we examined when Sam68 protein levels elevated during the course of spontaneous skin tumor development in *Gli2* ^tg/+^ mice, by examining the relative Sam68 protein levels in the ear tissues from wild‐type and *Gli2* ^tg/+^ mice at various periods after birth. Indeed, while the relative levels of Sam68 maintained comparable in WT mouse skin tissues ranging from P20 to P100, they substantially increased in *Gli2* ^tg/+^ mouse ear skin at P40 and such elevation was more profound at P60 and thereafter (Figure 1E). These results suggest that the elevated Sam68 protein levels correlate with, and thus could contribute to, the development of human and mouse NMSC.

### 3.2 Sam68 is crucial for the growth and survival of human skin cancer cells

**Figure 2:**
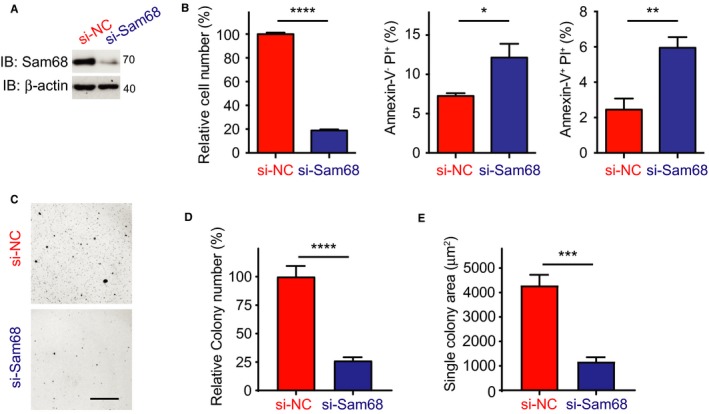
Sam68 is essential for survival and growth of skin cancer cells. A, A431 cells were transfected with nonspecific control (si‐NC) or Sam68‐specific (si‐Sam68) small interference RNAs. Seventy‐two hours later, whole cell lysates were derived and immunoblotted (IB) for Sam68, with β‐actin as a loading control. B, A431 cells expressing si‐NC or si‐Sam68 siRNAs were stained by propidium iodide (PI) and Annexin V, followed by flow cytometry analysis. Percentages of relative cell numbers, cells in early apoptosis (PI^−^ Annexin V^+^), and late apoptosis (PI^+^ Annexin V^+^) were quantified, respectively. C, Representative microphotographs of anchorage‐independent growth of A431 cells expressing either si‐NC or si‐Sam68 siRNAs, taken 3 weeks post siRNA transfection. Scale bars, 5 mm. Each experiment contained at least three replicates per condition, and every experiment was performed at least three times. D‐E, Quantification of anchorage‐independent grown colony numbers (D) and areas (E) of A431 cells, as in (C), from six random fields. Data information: In (B, D‐E), data are presented as mean ± SEM. *P < .05; **P < .01; ***P < .001; ****P < .0001 (Student's *t* test)

To assess the impact of Sam68 on the cell growth and survival of NMSC, we downregulated Sam68 expression in A431 cells, a human malignant keratinocyte cell line, using small interference RNAs (siRNAs). As expected, Sam68‐specific siRNA transfection significantly reduced Sam68 level in A431 cell (Figure 2A). Interestingly, Sam68 knockdown, in comparison to nonspecific controls, substantially sensitized A431 cells to undergo programmed cell death spontaneously, with increased percentages of early (Annexin V^+^PI^−^) and late (Annexin V^+^PI^+^) apoptotic cells, as analyzed by Annexin V and propidium iodide (PI) staining (Figure 2B). To further assess the impact of Sam68 on the oncogenic transformation properties of A431 cells, we carried out anchorage‐independent colony formation (soft agar) assays, which have been widely used to evaluate the loss of contract inhibition of growth, a hallmark of cancer cells.^25^ Substantially less colonies that otherwise showed reduced growth areas grew within the soft agar from A431 cells expressing Sam68‐specific siRNA compared to the nonspecific controls (Figure 2C‐E). Application of the EdU cell proliferation assay showed that knockdown of Sam68 has no significant influence on proliferation of A431 cells (Figure S1). Taken together, these results suggest that Sam68 plays an important role in the growth and survival of human NMSC cells.

### 3.3 Sam68 is essential for mouse skin tumor development and survival

**Figure 3:**
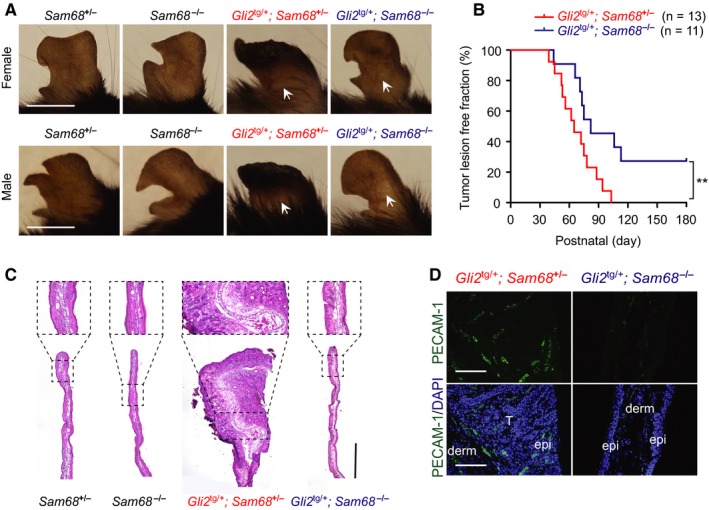
Sam68 plays a critical role for mouse development of skin cancer. A, Representative macrographs of P70 ears from the mice with indicated genders and genotypes. Arrows point to blood vessels. Scale bars, 1 cm. B, The tumor lesion onset kinetics in *Gli2* ^tg/+^;*Sam68* ^±^ and *Gli2* ^tg/+^;*Sam68* ^−/−^ mice. **P < .02 by Gehan‐Breslow‐Wilcoxon test. C, Hematoxylin and eosin staining of P70 ear tissue sections derived from mice with indicated genotypes. Scale bar, 1 mm. D, Immunofluorescence micrographs of PECAM‐1, with nuclei counterstained by DAPI, on P70 ear tissue sections from *Gli2* ^tg/+^;*Sam68* ^±^ and *Gli2* ^tg/+^;*Sam68* ^−/−^ mice. T, tumor; derm, dermis; epi, epidermis. Scale bars, 100 μm. Data information: In (B), **P < .02 (Gehan‐Breslow‐Wilcoxon test)

To assess the impact of Sam68 in skin tumor development and survival, we next examined onset of ear lesions and epidermal hyperplasia in *Gli2* ^tg/+^ mice in the presence and absence of Sam68. Consistent with our previous report,^20^ lesions arise with complete penetrance between P45 and P180 in *Gli2* ^tg/+^; *Sam68* ^±^ mouse ear skin, but with no apparent discordance between the sexes (Figure 3A,B). In comparison to *Gli2* ^tg/+^; *Sam68* ^±^ mice, *Gli2* ^tg/+^; *Sam68* ^‐/‐^ mice exhibited a significant delay in the onset of ear skin lesions (Figure 3A,B). Moreover, the delay of skin tumorigenesis in *Gli2* ^tg/+^; *Sam68* ^‐/‐^ mice correlated with profound reductions in several key determinants of tumor growth, including morphology of ear tissue (Figure 3C) and blood vessel expansion (Figure 3A,D). Thus, these results suggest an essential role of Sam68 in the skin tumor growth and survival in *Gli2* ^tg/+^ mice.

### 3.4 Sam68 regulates DNA damage signaling pathways in skin cells

**Figure 4:**
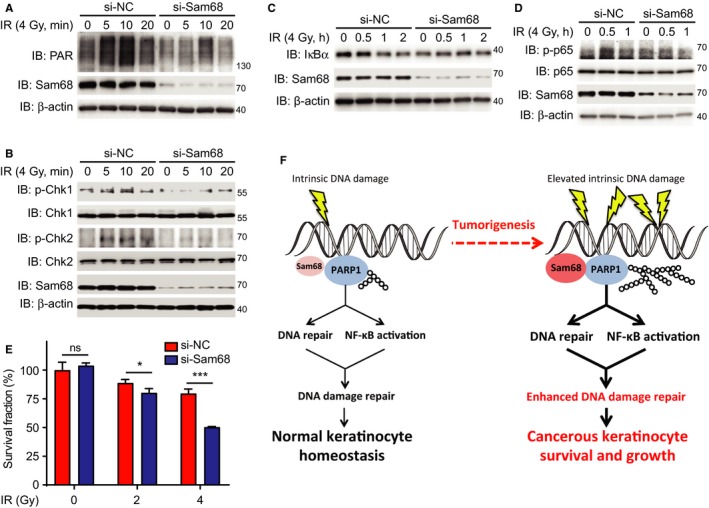
Sam68 is essential for DNA damage‐induced DNA repair and NF‐κB signaling pathways. A‐D, Immortalized keratinocytes were transfected with nonspecific control (si‐NC) or Sam68‐specific (si‐Sam68) small interference RNAs. Seventy‐two hours later, whole cell lysates were derived at the indicated periods post 4 Gy of γ‐irradiated (IR) and immunoblotted (IB) for indicated proteins, with β‐actin as a loading control. p‐Chk1, phosphorylated Chk1; p‐Chk2, phosphorylated Chk2; p‐p65, phosphorylated p65. E, Survival fraction of keratinocytes at 96‐h posttreatment with indicated doses of γ‐irradiation (IR). F, Schematic model representation of Sam68 functioning as regulator of DNA damage responses through controlling PAR synthesis in normal and cancerous keratinocytes. Data information: In (E), data are presented as mean ± SEM, *ns*, nonsignificant difference, and *P < .05; ***P < .001 (Student's *t* test)

We recently reported that Sam68 is an important early DNA damage signaling molecule that regulates DNA damage‐induced activation of poly(ADP‐ribose) polymerase 1 (PARP1) thus fulfilling an essential function in DNA repair and NF‐κB activation signaling pathways.^12^, ^13^, ^14^ As tumor cells generally acquire enhanced DNA repair activities to overcome the intrinsic DNA damage that frequently occurs during rapid proliferation, we sought to examine whether elevated levels of Sam68 in NMSC are essential for the cellular responses to DNA damage. As expected, γ‐irradiation (IR) triggered a robust PAR chain synthesis in immortalized mouse keratinocytes transfected with nonspecific control siRNA, peaking at 10 minutes posttreatment (Figure 4A). However, IR‐induced PAR chain formation was markedly dampened in the immortalized mouse keratinocytes with downregulated Sam68 (Figure 4A). Moreover, in comparison to controls, the phosphorylation of Chk1 and Chk2, two essential signaling transducers in cellular response to DNA damage,^26^ was attenuated in Sam68 knockdown keratinocytes (Figure 4B), supporting a key role of Sam68 in DNA repair signaling in response to DNA damage in keratinocytes.

We further examined the impact of Sam68 on NF‐κB activation signaling pathway, another important mediator of cellular responses to DNA damage.^12^, ^14^ As expected, IR triggered IκBα degradation, which is a prerequisite for the nuclear translocation of p65, in a time‐dependent manner in control keratinocytes (Figure 4C). In contrast, DNA damage‐induced IκBα degradation was attenuated in Sam68 knockdown keratinocytes (Figure 4C). Consistently, phosphorylated p65 (p‐p65), another biochemical hallmark of NF‐κB activation, was also tempered in Sam68 knockdown keratinocytes in comparison to control cells (Figure 4D). These results hence suggest that Sam68 is essential for DNA damage‐induced NF‐κB activation signaling pathway in skin keratinocytes. To assess the relevance of DDR deficiency caused by Sam68 knockdown, we examined the effect of Sam68 knockdown on clonogenic survival of mouse keratinocytes following exposure to IR. Indeed, Sam68 knockdown in keratinocytes led to an increased sensitivity to IR, compared with control cells (Figure 4E). Altogether, our results suggest that Sam68 is pivotal for DNA damage‐initiated repair and NF‐κB signaling pathways thus conferring the sensitivity of keratinocytes to DNA damaging agents (Figure 4F).

## 4 DISCUSSION

Since its original identification as the first mitotic substrate and binding partner of Src tyrosine kinase,^27^, ^28^ Sam68 has been reported to fulfill versatile functions in an array of important cellular processes including RNA metabolism, adipogenesis, gametogenesis, transcription, and others.^29^ More importantly, an increasing number of studies support an emerging role of Sam68 in tumor progression and development 14, 30, 31, 32, 33; in particular, we recently uncovered that Sam68 is a key regulator of the DNA damage sensor PARP1 and plays an indispensable role in the very early cellular signaling cascade in response to DNA damage.^13^ Herein, we report that Sam68 regulates DNA damage‐initiated DNA repair and NF‐κB signaling pathways in keratinocytes and that Sam68 is crucial for the growth and survival of skin tumors in mice, which suggests a novel role of Sam68 in the development of NMSC and lends additional support to the pro‐oncogenic properties of Sam68. Our current work has added NMSC to the wide spectrum of cancers in which Sam68 expression is significantly elevated and correlates with tumor progression, including colon cancer,^14^, ^18^ adult myeloid leukemia,^32^ oral tongue cancer,^15^ breast cancer,^16^ renal cell carcinoma,^17^ and now, nonmelanoma skin cancer (this study). The evidence herein reported suggests that Sam68, given a ubiquitous expression pattern, could serve as a general prognostic marker for multiple cancers. Future efforts should be devoted to decipher the full significance and mechanisms involved in relating Sam68 to cancer development.

DNA damage constantly occurs in the cell, and accordingly, the cellular DDR machinery has evolved to deal effectively with this challenge.^34^ Cancer cells possess a greater propensity to accumulate DNA damage, owing to massive intrinsic DNA damage that occurs during rapid DNA replication and cell proliferation; hence, cancer cells generally acquire hyperactivated DDR machinery and elevated DNA repair capability.^25^ Frequently, traditional cancer treatments entail chemotherapy and radiotherapy that target DDR in cancer cells; however, such approaches can also result in damage to normal tissues and unwanted side effects.^7^ To kill cancer cells specifically and effectively, it might prove ideal to target the unique abnormalities in the DDR machinery that specifically exists in cancer cells but not healthy normal cells. As a key early signaling regulator at the proxy of the nuclear‐initiated DNA repair and NF‐κB signaling pathway,^12^, ^13^, ^14^ Sam68 could provide a novel target for therapeutics more selective for cancers. Here, we report that Sam68 knockdown sensitizes human malignant keratinocytes to DNA damaging agents and Sam68 deletion markedly retards skin tumor burden and survival in *Gli2* ^tg/+^ mice. These results indicate that Sam68 could potentially be a novel therapeutic target for the treatment of NMSC.

## CONFLICT OF INTEREST

The authors have no conflict of interest to report.

## AUTHOR CONTRIBUTIONS

KF, XS, PAC, and FW designed the experiments. KF and XS conducted most experiments, acquisition of data, and contributed equally. XX, YG, and RPH helped with some experiments. All authors contributed to analysis and interpretation of data. KF and FW wrote the manuscript with input from all authors.

## References

[1] Stern RS . Prevalence of a history of skin cancer in 2007: results of an incidence‐based model. Arch Dermatol. 2010;146(3):279‐282. 20231498 10.1001/archdermatol.2010.4

[2] Rogers HW , Weinstock MA , Feldman SR , Coldiron BM . Incidence estimate of nonmelanoma skin cancer (keratinocyte carcinomas) in the U.S. Population, 2012. JAMA Dermatol. 2015;151(10):1081‐1086. 25928283 10.1001/jamadermatol.2015.1187

[3] Tang L , Chen X , Zhang XU , et al. N‐Glycosylation in progression of skin cancer. Med Oncol. 2019;36(6):50. 31037368 10.1007/s12032-019-1270-4

[4] Minocha R , Damian DL , Halliday GM . Melanoma and nonmelanoma skin cancer chemoprevention: a role for nicotinamide? Photodermatol Photoimmunol Photomed. 2018;34:5‐12. 28681504 10.1111/phpp.12328

[5] Katiyar SK , Pal HC , Prasad R . Dietary proanthocyanidins prevent ultraviolet radiation‐induced non‐melanoma skin cancer through enhanced repair of damaged DNA‐dependent activation of immune sensitivity. Semin Cancer Biol. 2017;46:138‐145. 28412456 10.1016/j.semcancer.2017.04.003

[6] Freitas AA , de Magalhaes JP . A review and appraisal of the DNA damage theory of ageing. Mutat Res. 2011;728(1–2):12‐22. 21600302 10.1016/j.mrrev.2011.05.001

[7] O'Connor MJ . Targeting the DNA damage response in cancer. Mol Cell. 2015;60(4):547‐560. 26590714 10.1016/j.molcel.2015.10.040

[8] Jackson SP , Bartek J . The DNA‐damage response in human biology and disease. Nature. 2009;461(7267):1071‐1078. 19847258 10.1038/nature08467 PMC2906700

[9] Ciccia A , Elledge SJ . The DNA damage response: making it safe to play with knives. Mol Cell. 2010;40(2):179‐204. 20965415 10.1016/j.molcel.2010.09.019 PMC2988877

[10] Rodríguez MI , Peralta‐Leal A , O'Valle F , et al. PARP‐1 regulates metastatic melanoma through modulation of vimentin‐induced malignant transformation. PLoS Genet. 2013;9(6):e1003531. 23785295 10.1371/journal.pgen.1003531 PMC3681683

[11] Czyż M , Toma M , Gajos‐Michniewicz A , et al. PARP1 inhibitor olaparib (Lynparza) exerts synthetic lethal effect against ligase 4‐deficient melanomas. Oncotarget. 2016;7(46):75551‐75560. 27705909 10.18632/oncotarget.12270 PMC5342760

[12] Fu K , Sun X , Wier EM , Hodgson A , Hobbs RP , Wan F . Sam68/KHDRBS1‐dependent NF‐kappaB activation confers radioprotection to the colon epithelium in gamma‐irradiated mice. Elife. 2016;5:e21957. 27996939 10.7554/eLife.21957 PMC5214542

[13] Sun X , Fu K , Hodgson A , et al. Sam68 is required for DNA damage responses via regulating poly(ADP‐ribosyl)ation. PLoS Biol. 2016;14(9):e1002543. 27635653 10.1371/journal.pbio.1002543 PMC5026359

[14] Fu K , Sun X , Wier EM , et al. Sam68/KHDRBS1 is critical for colon tumorigenesis by regulating genotoxic stress‐induced NF‐kappaB activation. Elife. 2016;5:e15018. 27458801 10.7554/eLife.15018 PMC4959885

[15] Chen SW , Zhang Q , Yang AK , et al. Overexpression and cytoplasmic localization of Sam68 correlate with tumour progression and poor prognosis in patients with clinically N0 oral tongue cancer. Head Neck Oncol. 2012;4(2):61.

[16] Song L , Wang L , Li Y , et al. Sam68 up‐regulation correlates with, and its down‐regulation inhibits, proliferation and tumourigenicity of breast cancer cells. J Pathol. 2010;222(3):227‐237. 20662004 10.1002/path.2751

[17] Zhang Z , Li J , Zheng H , et al. Expression and cytoplasmic localization of SAM68 is a significant and independent prognostic marker for renal cell carcinoma. Cancer Epidemiol Biomarkers Prev. 2009;18(10):2685‐2693. 19755649 10.1158/1055-9965.EPI-09-0097

[18] Liao W‐T , Liu J‐L , Wang Z‐G , et al. High expression level and nuclear localization of Sam68 are associated with progression and poor prognosis in colorectal cancer. BMC Gastroenterol. 2013;13(1):126. 23937454 10.1186/1471-230X-13-126 PMC3751151

[19] Richard S , Torabi N , Franco GV , et al. Ablation of the Sam68 RNA binding protein protects mice from age‐related bone loss. PLoS Genet. 2005;1(6):e74. 16362077 10.1371/journal.pgen.0010074 PMC1315279

[20] Hobbs RP , DePianto DJ , Jacob JT , et al. Keratin‐dependent regulation of Aire and gene expression in skin tumor keratinocytes. Nat Genet. 2015;47(8):933‐938. 26168014 10.1038/ng.3355 PMC4520766

[21] Reichelt J , Haase I . Establishment of spontaneously immortalized keratinocyte lines from wild‐type and mutant mice. Methods Mol Biol. 2010;585:59‐69. 19907996 10.1007/978-1-60761-380-0_5

[22] Fu K , Sun X , Zheng W , et al. Sam68 modulates the promoter specificity of NF‐kappaB and mediates expression of CD25 in activated T cells. Nat Commun. 2013;4:1909. 23715268 10.1038/ncomms2916 PMC3684077

[23] Hodgson A , Wier EM , Fu K , et al. Metalloprotease NleC suppresses host NF‐kappaB/Inflammatory responses by cleaving p65 and interfering with the p65/RPS3 interaction. PLoS Pathog. 2015;11(3):e1004705. 25756944 10.1371/journal.ppat.1004705 PMC4355070

[24] Grachtchouk M , Mo R , Yu S , et al. Basal cell carcinomas in mice overexpressing Gli2 in skin. Nat Genet. 2000;24(3):216‐217. 10700170 10.1038/73417

[25] Hanahan D , Weinberg RA . Hallmarks of cancer: the next generation. Cell. 2011;144(5):646‐674. 21376230 10.1016/j.cell.2011.02.013

[26] Polo SE , Jackson SP . Dynamics of DNA damage response proteins at DNA breaks: a focus on protein modifications. Genes Dev. 2011;25(5):409‐433. 21363960 10.1101/gad.2021311 PMC3049283

[27] Taylor SJ , Shalloway D . An RNA‐binding protein associated with Src through its SH2 and SH3 domains in mitosis. Nature. 1994;368(6474):867‐871. 7512694 10.1038/368867a0

[28] Fumagalli S , Totty NF , Hsuan JJ , Courtneidge SA . A target for Src in mitosis. Nature. 1994;368(6474):871‐874. 7512695 10.1038/368871a0

[29] Richard S . Reaching for the stars: linking RNA binding proteins to diseases. Adv Exp Med Biol. 2010;693:142‐157. 21189691

[30] Li N , Ngo CT , Aleynikova O , Beauchemin N , Richard S . The p53 status can influence the role of Sam68 in tumorigenesis. Oncotarget. 2016;7(44):71651‐71659. 27690217 10.18632/oncotarget.12305 PMC5342108

[31] Li N , Richard S . Sam68 functions as a transcriptional coactivator of the p53 tumor suppressor. Nucleic Acids Res. 2016;44(18):8726‐8741. 27365047 10.1093/nar/gkw582 PMC5062974

[32] Benoit YD , Mitchell RR , Risueño RM , et al. Allows selective targeting of human cancer stem cells. Cell Chem Biol. 2017;24(7):833–844.e9. 28648376 10.1016/j.chembiol.2017.05.026

[33] Fu K , Wan F . Sam68 offers selectively aimed modulation of transcription in cancer stem cells. Cell Chem Biol. 2017;24(7):777‐779. 28732196 10.1016/j.chembiol.2017.07.003 PMC5909376

[34] Hosoya N , Miyagawa K . Targeting DNA damage response in cancer therapy. Cancer Sci. 2014;105(4):370‐388. 24484288 10.1111/cas.12366 PMC4317796

