# Supplementary material for: Sam68 is required for the growth and survival of nonmelanoma skin cancer
Source: Cancer Med. 2019 Aug 22;8(13):6106–13. doi: 10.1002/cam4.2513 (PMC6792479; doi:10.1002/cam4.2513)
Supplement: Supplementary file 1 [file CAM4-8-6106-s001.docx]

**
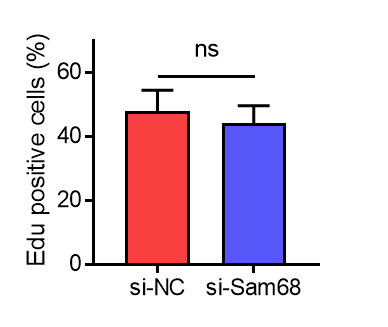

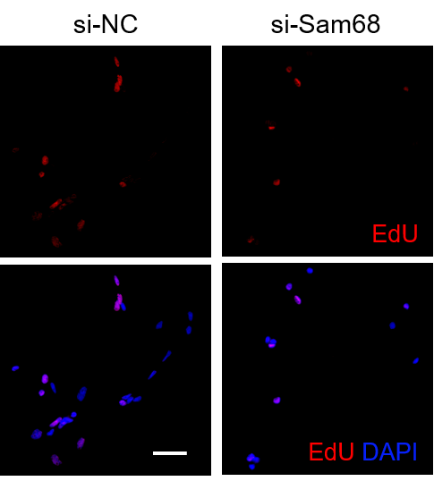
A B**

**SUPPLEMENTARY FIGURE 1.** Sam68 is not required for the proliferation of A431 cells. (A) The Click-iT EdU cell proliferation assay (Invitrogen) for detection of replicating cells is based on incorporation of EdU into newly synthesized DNA. Briefly, after 72 hours knockdown of Sam68 in A431 cells, EdU containing culture media was added. The cells were fixed by 3.7% formaldehyde and permeabilized by 0.5% Triton after 4 hours culture, then incubated with Click-iT reaction cocktail. After staining, total DNA was detected with DAPI and coverslips were mounted before analysis by fluorescence microscopy. Scale bar, 50 μm. (B) Percentage of EdU^+^ (from 5 random fields) cells was quantified from (A). Data are presented as mean ± s.e.m, ns, non-significant difference (Student’s t test).
